# Utilizing Next-Generation Sequencing: Advancements in the Diagnosis of Fungal Infections

**DOI:** 10.3390/diagnostics14151664

**Published:** 2024-08-01

**Authors:** Sheetal Naik, Dharambir Kashyap, Jashan Deep, Saif Darwish, Joseph Cross, Edmond Mansoor, Vivek Kumar Garg, Prasanna Honnavar

**Affiliations:** 1 Department of Physiology, American University of Antigua College of Medicine, St. Johns 1451, Antigua and Barbuda; snaik@auamed.net; 2 Brown Center for Immunotherapy, Melvin and Bren Simon Comprehensive Cancer Center, Division of Hematology and Oncology, School of Medicine, Indiana University, Indianapolis, IN 46202, USA; dbir@iu.edu; 3 Basic Medical Science, American University of Antigua College of Medicine, St. Johns 1451, Antigua and Barbuda; 4 Department of Biochemistry, Cell Biology and Genetics; American University of Antigua College of Medicine, St. Johns 1451, Antigua and Barbuda; 5 Department of Microbial Pathogenesis and Immunology, Texas A & M University, College Station, TX 77843, USA; 6 Department of Clinical Medicine; American University of Antigua College of Medicine, St. Johns 1451, Antigua and Barbuda; 7 University Institute of Allied Health Sciences, Chandigarh University, Gharuan, Mohali 140413, Punjab, India; vivek.e10915@cumail.in; 8 Department of Microbiology and Immunology; American University of Antigua College of Medicine, St. Johns 1451, Antigua and Barbuda

**Keywords:** next-generation sequencing, fungal infection, diagnosis, pneumonia

## Abstract

Next-generation sequencing (NGS) has emerged as a promising tool for diagnosing fungal infections. It enables the identification of a wide range of fungal species and provides more accurate and rapid results than traditional diagnostic methods. NGS-based approaches involve the sequencing of DNA or RNA from clinical samples, which can be used to detect and identify fungal pathogens in complex clinical samples. The development of targeted gene panels and whole-genome sequencing has allowed for identifying genetic markers associated with antifungal drug resistance, enabling clinicians to tailor patient treatment options. NGS can also provide insights into the pathogenesis of fungal infections and aid in discovering novel drug targets. Although NGS has some limitations, such as cost and data analysis, it can potentially revolutionize the future diagnosis and treatment of fungal infections.

## 1. Introduction

The ultimate goal of the clinical microbiology laboratory is to diagnose pathogenic organisms rapidly and accurately. Rapid and accurate diagnoses assure successful treatment and better patient outcomes. Infectious and communicable diseases are one of the leading causes of death worldwide, especially in low-income countries. Accurate and rapid diagnosis of contagious disease-causing pathogens is the need of the hour in clinical microbiology laboratories and the public health sectors [1]. As per the Global Action Fund for fungal infections (GAFFI-https://gaffi.org/, accessed on 4 June 2024), over 300 million individuals worldwide suffer from serious fungal infections, resulting in over 1.5 million deaths yearly [2]. More than 25 million have life-threatening infections or run the risk of blindness. Fungal infections can be chronic, such as chronic pulmonary aspergillosis or tinea capitis; recurrent oral thrush in AIDS patients and candida vaginitis; or acute and severe, such as keratitis and cryptococcal meningitis.

At the global level, estimates show that among AIDS patients, Pneumocystis pneumonia and Cryptococcal meningitis have affected >400,000 and 223,000 individuals, respectively, worldwide [2]. Both have a case fatality rate of 15%. Fungal keratitis has affected ~1.4 million people, resulting in >600,000 cases of blindness [3]. Estimated deaths from invasive infections, including *Aspergillus* sp., *Candida* sp., and *Histoplasma* sp., are >500,000, >350,000, and >80,000, respectively [4]. Severe asthma with fungal sensitization (SAFS) and chronic pulmonary aspergillosis have resulted in the death of 500,000 and >450,000, respectively. Taken together, ~13.5 million people have been affected with fungal infections, leading to >1.6 million deaths. These numbers are probably underestimates (https://gaffi.org/, accessed on 4 June 2024). 

Pathogenic fungi can be identified by conventional/traditional/non-DNA sequence-based methods and DNA sequence-based techniques. Most routine fungal diagnostic laboratories use Sabouraud dextrose/potato dextrose media for fungal culture and lactophenol cotton blue to stain the mycelia. Despite the availability of advanced molecular diagnostic tools, conventional techniques still have an essential role in routine clinical fungal diagnostics. However, conventional methods are more time-consuming, provide low positivity rates, and do not adequately meet clinical needs, such as the rapid diagnosis required for invasive fungal infections (IFIs) in immunocompromised patients [5]. 

After the growth on the culture media, fungal pathogens can be further identified or characterized based on their biochemical properties or by PCR amplification of the Internal Transcribed Spacer region (ITS) or D1/D2 domain of large subunit ribosomal DNA (yeasts/molds). The matrix-assisted laser desorption ionization time-of-flight mass spectrometry (MALDI-TOF MS) technique has been proven to be a reliable and high-throughput tool for fungus identification. However, prerequisites of fungal growth and a mass spectra reference database are the limiting factors of MALDI-TOF [6]. Some fungi strains are very slow growers and take up to 4 weeks to grow [7]. 

New advancements in molecular biology have resulted in several new diagnostic techniques in the 21st century, such as Enzyme-Linked Immunosorbent Assay (ELISA)-based hybridization assay, fluorescently labeled real-time detection, solid and liquid phase microarray, pan fungal Polymerase Chain Reaction (PCR), Random Amplified Polymorphic DNA (RAPD), PCR-Restriction Fragment Length Polymorphism (PCR-RFLP), DNA/RNA hybridization probes, PCR fingerprinting, real-time PCR assays, molecular typing like Multilocus Sequence Typing (MLST), Multilocus Microsatellite Typing (MLMT), and Amplified Fragment Length Polymorphism (AFLP). DNA Sanger sequencing has dominated molecular diagnostics for the last two decades [8,9]. A detailed history of the discovery of molecular diagnostics techniques and their applications is provided in Table 1.

Next-generation sequencing (NGS) tools such as metabarcoding (targeted amplicon sequencing) and shotgun metagenomics have revolutionized the field of molecular biology. The NGS has the potential to accurately identify pathogens that are difficult to culturable, rare, and previously unknown in even a tiny quantity of clinical samples. The advantages and utilization of NGS in clinical diagnosis have been explored remarkably. This high throughput technique not only identifies but can also determine the abundance of microorganisms such as bacteria, viruses, fungi, parasites, and other atypical pathogens in clinical samples, including cerebrospinal fluid (CSF), bronchoalveolar lavage (BAL), sputum, and whole blood [10]. The NGS is a rapid and more accurate tool that does not require fungi to be cultured for identification and can distinguish different strains in mixed infections. Long-run sequences can even be used to identify the antimicrobial resistance profile. Metagenomic NGS (mNGS) applications have been applied for mycobiome analysis and identification from the soil, water, air, human skin, gut, and veterinary samples. Over 200 fungus species have been identified and categorized from the human digestive tract [11]. The application of NGS in diagnosing fungal infections compared to bacterial infections in humans is currently limited. The current review article summarizes the applications of NGS tools in fungus diagnostics and their future perspective. 

## 2. Next-Generation Sequencing over Conventional Testing

Conventional testing (CNT) requires low-cost laboratory equipment and reagents compared to the NGS setup. The cost of testing using conventional methods is lower than that of NGS runs. However, sample testing costs in NGS have been drastically reduced (Tsang and 2021). The sequencing cost has even been reduced to 100 USD. Compared to CNT, which involves manual interpretation and handling of samples, NGS involves sophisticated or computational sample preparation, running equipment, and data interpretation [9].

CNT and NGS require similar time frames for sample collection, which typically range from a few minutes to a few hours, depending on the sample type and location. CNT usually requires 1–2 days for sample preparation, which includes culturing and incubation. NGS also requires 1–2 days for sample preparation, which provides for DNA extraction and library preparation. CNT can take anywhere from a few hours to two weeks to produce results, depending on the type of test and microorganism being detected, and in some situations, we may not get definitive results [12]. NGS can take 6 h to 7 days to generate results but can produce a more comprehensive analysis of microbial communities. The turnaround time to generate NGS results depends on the sequencing technologies and bioinformatics involved [13]. NGS is best suited for diagnosing fungal infection because of low fungal load, difficulty in culturing, and slow growth rate [14]. Overall, CNT has a faster turnaround time than NGS for routine testing. Still, NGS may be more suitable for specific applications where a more comprehensive analysis is required, and a longer turnaround time is acceptable. 

CNT is generally considered highly accurate when performed correctly, but it may not detect all microorganisms present in a sample. NGS can provide a more precise and comprehensive analysis of microbial communities, but it is still subject to potential errors and biases in sample preparation, sequencing, and bioinformatics analysis [12]. CNT results are typically interpreted by trained microbiologists or clinical laboratory personnel, who may have varying experience levels and expertise. NGS results require specialized bioinformatics analysis to analyze the large amount of sequencing data generated, which may require additional knowledge and training [15]. CNT results often directly apply to clinical decision-making, such as selecting appropriate antimicrobial therapy. NGS results may provide more detailed information on microbial communities but may require additional interpretation and integration with clinical data to be directly applicable to clinical decision-making. Overall, while CNT remains the gold standard for routine clinical microbiology testing, NGS has the potential to provide a more comprehensive and accurate analysis of microbial communities but may require additional expertise and interpretation to be directly applicable to clinical decision-making [9]. Table 2 summarizes the comparison between CNT and NGS.

## 3. Application of NGS in Diagnoses of Fungal Infection

**Figure 1:**
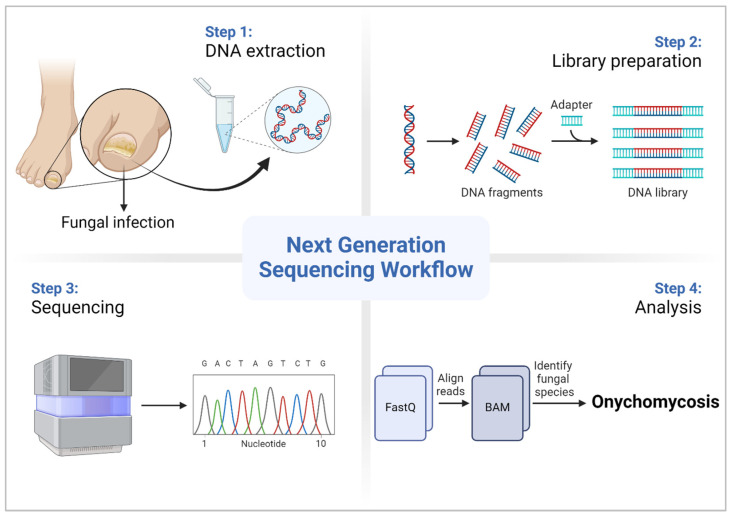
Next-generation Sequencing workflow for fungal diagnosis (created using biorender.com).

Compared to the NGS application for bacteria and virus detection, clinical fungal detection is sparse due to only a few hundred NGS fungal genome sequences [10,16]. NGS technology has diagnosed ~300 fungal infection cases till now [14] (Figure 1). In a retrospective study conducted at Shanghai Pulmonary Hospital, China, NGS detected 95% of bacterial and fungal infections, whereas the ‘gold standard’ culture method detected only 60% in 20 pre-diagnosed patient samples [17]. In a study conducted by Wu et al., mNGS detected 29 *Pneumocystis jirovicii* infections, whereas Wright-Giemsa-stained smear detected only 8 *Pneumocystis jirovicii*. The specificity of mNGS was 100%, whereas the G test [(1→3)-β-D-glucan test] combined with serum lactate dehydrogenase had only 56% [18]. In a study by Paige et al., NGS identified microorganisms in 100% (44/44) of histopathologically positive formalin-fixed, paraffin-embedded tissues. In contrast, culture-based methods had identification in 27.3% (12/44) of samples [19]. Using NGS, Doan et al. identified *Candida sublicenses* in previously diagnosed negative (8/36) uveitis archived intraocular fluid samples [20]. The application of mNGS in Candidiasis is not practical. However, it has been used in difficult-to-diagnose cases of *C. tropicalis* [21] and *C. sublicenses* [22]. mNGS has also been used to diagnose cryptococcal meningitis and cryptococcal osteomyelitis [23,24]. Histoplasmosis diagnosis and prognosis can be monitored by mNGS tools [25]. This high throughput technique has also been used to diagnose *Aspergillus fumigatus* in BAL samples of non-neutropenic invasive pulmonary aspergillosis cases and *A. flavus* from endocarditis [26,27].

NGS was used to reveal the presence of fungi in brain samples of Alzheimer’s and Huntington’s disease [28,29,30]. The intensity of fungal detection was higher using NGS than the CNT while investigating the ocular surface (73.5% vs. 12.5%) [31] and endophthalmitis [32]. Imabayashi et al. identified 67 fungal species predominated with *C. albicans* in 27 oral candidiasis patients using NGS. The NGS was also used to report fungi species composition in patients before and after antifungal therapy [33]. Further, by application of NGS, Malassezia spp. has been determined as a unique biomarker to distinguish oral squamous cell carcinoma patients from healthy individuals [34]. NGS technology has shown that pulmonary exacerbation in cystic fibrosis was related to *Aspergillus* and *Malassezia* spp., and reduction in lung function was associated with *Scedosporium* sp. [35]. Diagnosing bloodstream infection (BSI) due to fungi is extremely challenging for CNT and NGS. Although fungi account for 30% of total BSI, CNT can detect only 2–3% [36]. Few studies have shown promising results in diagnosing fungal BSI by NGS application [37,38,39,40].

## 4. Metagenomics and Metabarcoding

Metagenomics has been extensively used in detecting and surveilling emerging and recurrent plant pathogens. The low precision of metagenomic can be overcome by choosing accurate primers and advanced bioinformatic algorithms. Metabarcoding is based on amplifying a well-characterized genetic region, whereas shotgun metagenomics identifies the sequences of the entire genetic content in a sample [9]. Metabarcoding differs from DNA barcoding in that it involves the simultaneous identification of multiple species within an environmental sample using high-throughput sequencing of a standardized genetic marker. In contrast, barcoding typically focuses on identifying a single species by sequencing a specific marker gene from an individual organism. Metabarcoding has several advantages, such as tiny DNA quantity for the procedure, high resolution, taxonomic coverage, and easy and fast data analysis. It is well suited for microbiome studies where multiple species can be identified and quantified simultaneously without culturing the sample. ITS1/2 region will be targeted in a mycobiome study. The DNA barcode can be queried against a reference database such as the International Society for Human and Animal Mycology (ISHAM), Reference Sequence (RefSeq), The Barcode of Life Data System (BOLD), and UNITE. However, it can only amplify short DNA sequences up to 500 bp to 600 bp and cannot resolve all species. Moreover, shotgun metagenomics identifies microorganisms and can characterize their genome profiling for antimicrobial resistance (AMR), genetic subtypes, epidemiological studies, outbreak tracing, and virulence [14]. The DNA/RNA extracted from the sample without culturing will undergo in-depth sequencing after library preparation and can sequence up to 10 kb of region. However, running costs are higher, data analysis is comprehensive, and subtracting the host or background DNA is very complex. It is challenging in non-sterile patient samples or co-infection. The length of DNA fragment sequencing depends on the quality and quantity of DNA. Mycobiota constitutes only 0.1–1% of the total microbiome compared to other microbiota. Therefore, successful shotgun metagenomics requires at least 1012 to 1014 nucleotides to be sequenced per run/sample. This is practically very challenging [9].

As per methodological and analytical differences considered, metabarcoding targets specific regions of the fungal genome (18S rRNA or ITS regions), while metagenomics, on the other hand, can sequence the entire genome of fungi. Metabarcoding uses PCR amplification to target the specific DNA regions of interest and may meet biases and errors during analysis. Metagenomics does not require PCR amplification but can provide a more comprehensive analysis of the fungi. Metabarcoding typically generates millions of reads, while metagenomics can generate up to billions of reads per sample [9]. This difference in sequence depth can impact the detection of rare or low-abundance microorganisms. Metabarcoding data analysis often involves clustering sequences into operational taxonomic units (OTUs) and assigning taxonomy based on a reference database. Metagenomics data analysis consists of assembling the reads into contiguous sequences and assigning taxonomy based on sequence homology to known genomes [14]. Both metagenomics and metabarcoding have strengths and weaknesses in analyzing microbial communities, and the choice of method depends on the research question and sample type. Metabarcoding is a more targeted approach that provides high taxonomic resolution for specific regions of interest. In contrast, metagenomics provides a more comprehensive analysis of the microbial community but may require more computational resources for analysis [9]. Metagenomics can identify an outbreak’s source and transmission routes by comparing the genetic makeup of the microbes in the infected patients to those in the environment or other sources. This approach can provide a comprehensive analysis of the microbial community and can detect rare or low-abundance microorganisms. In contrast, metabarcoding is more targeted and may not detect rare or low-abundance microorganisms, limiting its ability to trace outbreaks [9]. Metagenomics can provide information on the presence and potential function of virulence genes in the microbial community, which can help understand the microbes’ pathogenesis [14]. Metabarcoding, conversely, targets specific regions of the genome and may not capture the full complement of virulence genes in the microbial community. Metagenomics can comprehensively analyze the resistome, or the genes involved in antibiotic resistance, in the microbial community. It can even reveal the undescribed mutation leading to resistance [41]. This approach can help understand the prevalence and diversity of resistance genes and their potential transfer among different microbes. Metabarcoding, on the other hand, is not well suited for detecting resistance genes not targeted by the chosen primers. Table 3 highlights the difference between metabarcoding and metagenomics.

## 5. NGS for Liquid Biopsy

**Figure 2:**
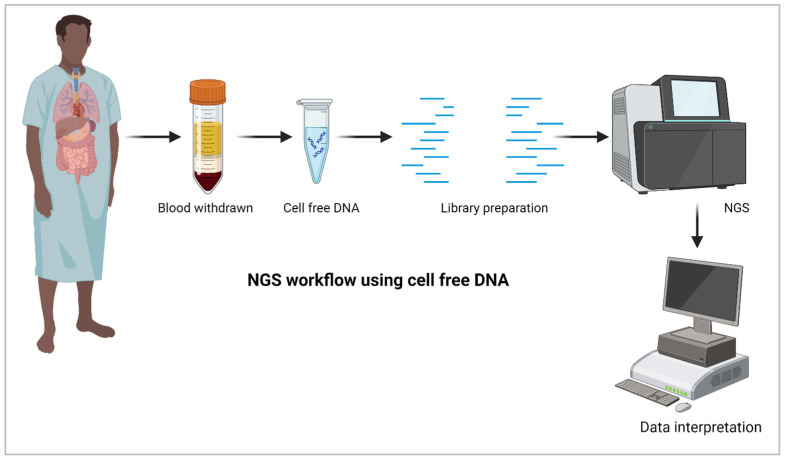
Illustration of NGS-based approaches for invasive fungal detection using cell-free DNA (created using biorender.com).

Liquid biopsies are non-invasive approaches for disease diagnosis, including fungal detection. Body fluids such as blood/plasma, milk, and urine can be used for clinical samples for liquid biopsy. These samples are processed for nucleic acid isolation, followed by NGS and fungal identification (Figure 2). Invasive fungal infection has been associated with fivefold increased mortality and millions of additional costs. The diagnostic evaluation includes extensive imaging to identify the source of the fever, have limited selectivity. The next-generation sequencing of cell-free plasma to detect pathogen DNA showed remarkable sensitivity [42,43]. Cell-free DNA is small fragments ~180 bp circulating in the blood. NGS-based analysis of these DNA fragments derived from fungal-infected dead and dying cells can be used successfully for diagnosis. NGS has been used to monitor transplant rejection and tumor tissues invaded by fungal spores. Infections in transplanted organs or tumors can be detected using cell-free DNA in blood [44].

## 6. Conclusions

NGS output gives massive data output. However, clinicians must receive condensed results without any unnecessary information to be helpful. NGS has the potential to be a game changer in diagnostics mycology. It could drastically reduce the time from diagnosis to treatment. NGS is an attractive molecular tool in diagnostics that needs to be evaluated in laboratory practice [45]. As most fungi are time-consuming to culture and identify through conventional means in lab conditions, NGS is a promising technique that can reduce the time to identification and hence deliver rapid, better treatment, leading to a better patient outcome.

## Figures and Tables

**Table 1:** A detailed journey of discovery of molecular techniques, description, and applications.

Decade	Technique	Description	Applications
1950s	Electrophoresis	Separation of molecules based on charge and size	Analysis of protein and nucleic acid molecules
1960s	Gel electrophoresis	Separation of DNA fragments by size	Analysis of genetic variation
1970s	DNA sequencing	Determination of nucleotide sequence of DNA	Analysis of genetic variation
1975	Southern blotting	Transfer of DNA fragments from a gel to a membrane for detection	Identification of specific DNA sequences
1980s	PCR	Amplification of specific DNA sequences	Analysis of specific regions of the genome
1983	DNA fingerprinting	Analysis of genetic variation using variable number tandem repeats (VNTRs)	Identification of individuals and populations
1985	DNA hybridization	Detection of complementary DNA sequences using labeled probes	Identification of specific DNA sequences
1986	DNA cloning	Insertion of a DNA fragment into a vector for replication and expression	Production of recombinant proteins and study of gene function
1990s	Fluorescent in situ hybridization (FISH)	Detection of specific DNA sequences in cells using fluorescently labeled probes	Visualization of microbial communities and identification of individual microbial species
1990s	18S rRNA/ITS sequencing (DNA barcoding)	Amplification and analysis of specific regions of the fungal genome	Identification of individual fungal species
1990s	Random amplified polymorphic DNA (RAPD)	Amplification of random regions of the genome for analysis of genetic variation	Identification of genetic markers and analysis of genetic diversity
1990s	Restriction fragment length polymorphism (RFLP)	Analysis of genetic variation based on restriction enzyme cleavage patterns	Identification of genetic markers and analysis of genetic diversity
1996	Differential display PCR	Analysis of differences in gene expression between cell populations	Identification of differentially expressed genes
2000s	Microarrays	Analysis of gene expression, SNP genotyping, and comparative genomic hybridization	Study of gene expression, detection of genetic variation, and identification of chromosomal abnormalities
2000s	Metagenomics	Comprehensive analysis of entire fungal communities	Identification of rare and uncultivable fungi
2000s	Metabarcoding	Comprehensive analysis of fungal communities based on barcode regions	Identification of rare and uncultivable fungi
Late 2000s	Next-generation sequencing (NGS)	Sequencing of entire genomes, transcriptomes, and epigenomes	Identification of genetic variation within and between fungal populations
Late 2000s	RNA sequencing	Analysis of gene expression and identification of novel transcripts	Study of gene regulation and identification of new genes
Recent years	Single-cell sequencing	Sequencing of individual microbial cells	Analysis of genomic variation at the single-cell level
Recent years	Metatranscriptomics	Analysis of gene expression in fungal communities	Study of fungal function and activity in different environments
Recent years	CRISPR (clustered regularly interspaced palindromic repeats)-Cas9	Targeted genome editing using RNA-guided endonucleases	Study of gene function and development of gene therapy treatments

**Table 2:** Comparison between conventional testing (CNT) and next-generation sequencing (NGS).

Criteria	Conventional Testing	Next-Generation Sequencing
Method	Culture-based methods, microscopy, and biochemical tests	High-throughput DNA sequencing
Sample types	Swabs, tissue samples, body fluids, food, environmental samples	DNA or RNA extracted from any sample
Detection limit	10^2^–10^3^ CFU/mL	1–10 CFU/mL
Speed of analysis	Hours to two weeks	Six h to 7 days
Level of detail	Limited identification to species level	Exact identification, including strain level
Sample size	Small to medium	Small to large
Cost	Low to moderate	High
Data output	Qualitative	Qualitative and quantitative
Data analysis	Manual interpretation required	Automated, computer-based analysis
Applications	- Routine clinical diagnostics - Food safety testing - Environmental monitoring - Antibiotic susceptibility testing	- Metagenomics and microbiome analysis - Pathogen discovery and characterization - Infectious disease diagnostics - Antimicrobial resistance and virulence factor detection - Environmental monitoring and bioremediation
Sensitivity and specificity	Moderate to high, depending on the method and organism	Very high
Advantages	- Well established and standardized - Relatively low cost - Detects viable organisms - Can identify antibiotic susceptibility	- Highly sensitive and specific - Detects viable and non-viable organisms - Provides detailed genetic information - Can identify multiple organisms simultaneously - Detects unculturable organisms
Disadvantages	- Limited to culturable organisms - Longer turnaround time - Difficulty in detecting mixed infections - Limited genetic information	- More expensive - Requires specialized equipment and expertise - Data interpretation can be complex - Cannot distinguish between living and dead pathogens as it may detect non-viable organisms

**Table 3:** Comparison between metabarcoding and metagenomics.

Parameter	Metabarcoding	Metagenomics
Target	Specific regions of the genome (18S rRNA or ITS regions for fungi)	The entire genetic content of the sample
PCR amplification	Yes	No
Sequence depth	Millions of reads per sample	Billions of reads per sample
Bias and errors	It may introduce biases and errors due to PCR amplification	Fewer biases and errors
Data Analysis	Clustering sequences into operational taxonomic units (OTUs) and assigning taxonomy based on a reference database	Assembling reads into contiguous sequences and assigning taxonomy based on sequence homology to known genomes.
Applications	Targeted analysis, studying specific taxonomic groups	Comprehensive analysis, identifying rare or low-abundance microorganisms, outbreak tracing, virulence detection, resistance detection

## Data Availability

Not applicable.

## References

[1] Fisher M.C., Denning D.W. (2023). The WHO fungal priority pathogens list as a game-changer. Nat. Rev. Microbiol..

[2] Bongomin F., Gago S., Oladele R.O., Denning D.W. (2017). Global and Multi-National Prevalence of Fungal Diseases-Estimate Precision. J. Fungi.

[3] Brown L., Leck A.K., Gichangi M., Burton M.J., Denning D.W. (2021). The global incidence and diagnosis of fungal keratitis. Lancet Infect. Dis..

[4] Rodrigues M.L., Nosanchuk J.D. (2020). Fungal diseases as neglected pathogens: A wake-up call to public health officials. PLoS Negl. Trop. Dis..

[5] Hoang M.T.V., Irinyi L., Hu Y., Schwessinger B., Meyer W. (2021). Long-Reads-Based Metagenomics in Clinical Diagnosis With a Special Focus on Fungal Infections. Front. Microbiol..

[6] Angeletti S. (2017). Matrix assisted laser desorption time of flight mass spectrometry (MALDI-TOF MS) in clinical microbiology. J. Microbiol. Methods.

[7] Irinyi L., Lackner M., de Hoog G.S., Meyer W. (2016). DNA barcoding of fungi causing infections in humans and animals. Fungal Biol..

[8] Burillo A., Bouza E. (2014). Use of rapid diagnostic techniques in ICU patients with infections. BMC Infect. Dis..

[9] Hilt E.E., Ferrieri P. (2022). Next Generation and Other Sequencing Technologies in Diagnostic Microbiology and Infectious Diseases. Genes.

[10] Jiang S., Chen Y., Han S., Lv L., Li L. (2022). Next-Generation Sequencing Applications for the Study of Fungal Pathogens. Microorganisms.

[11] Gouba N., Drancourt M. (2015). Digestive tract mycobiota: A source of infection. Med. Mal. Infect..

[12] Consortium O., Gabaldon T. (2019). Recent trends in molecular diagnostics of yeast infections: From PCR to NGS. FEMS Microbiol. Rev..

[13] Wu J., Lu A.D., Zhang L.P., Zuo Y.X., Jia Y.P. (2019). Study of clinical outcome and prognosis in pediatric core binding factor-acute myeloid leukemia. Zhonghua Xue Ye Xue Za Zhi.

[14] Tsang C.C., Teng J.L.L., Lau S.K.P., Woo P.C.Y. (2021). Rapid Genomic Diagnosis of Fungal Infections in the Age of Next-Generation Sequencing. J. Fungi.

[15] Gaston D.C., Miller H.B., Fissel J.A., Jacobs E., Gough E., Wu J., Klein E.Y., Carroll K.C., Simner P.J. (2022). Evaluation of Metagenomic and Targeted Next-Generation Sequencing Workflows for Detection of Respiratory Pathogens from Bronchoalveolar Lavage Fluid Specimens. J. Clin. Microbiol..

[16] Thomas R.S., Henson A., Gerrish A., Jones L., Williams J., Kidd E.J. (2016). Decreasing the expression of PICALM reduces endocytosis and the activity of beta-secretase: Implications for Alzheimer’s disease. BMC Neurosci..

[17] Chen P., Sun W., He Y. (2020). Comparison of the next-generation sequencing (NGS) technology with culture methods in the diagnosis of bacterial and fungal infections. J. Thorac. Dis..

[18] Wu X., Li Y., Zhang M., Li M., Zhang R., Lu X., Gao W., Li Q., Xia Y., Pan P. (2020). Etiology of Severe Community-Acquired Pneumonia in Adults Based on Metagenomic Next-Generation Sequencing: A Prospective Multicenter Study. Infect. Dis. Ther..

[19] Larkin P.M.K., Lawson K.L., Contreras D.A., Le C.Q., Trejo M., Realegeno S., Hilt E.E., Chandrasekaran S., Garner O.B., Fishbein G.A. (2020). Amplicon-Based Next-Generation Sequencing for Detection of Fungi in Formalin-Fixed, Paraffin-Embedded Tissues: Correlation with Histopathology and Clinical Applications. J. Mol. Diagn..

[20] Doan T., Acharya N.R., Pinsky B.A., Sahoo M.K., Chow E.D., Banaei N., Budvytiene I., Cevallos V., Zhong L., Zhou Z. (2017). Metagenomic DNA Sequencing for the Diagnosis of Intraocular Infections. Ophthalmology.

[21] Jin Y., Wang Z., Zhu C., Yang Q., Lu Y., Yu X., Hong B., Wang X., Zhang Y. (2021). Case Report: Proven Diagnosis of Culture-Negative Chronic Disseminated Candidiasis in a Patient Suffering From Hematological Malignancy: Combined Application of mNGS and CFW Staining. Front. Med..

[22] Wilson M.R., O’Donovan B.D., Gelfand J.M., Sample H.A., Chow F.C., Betjemann J.P., Shah M.P., Richie M.B., Gorman M.P., Hajj-Ali R.A. (2018). Chronic Meningitis Investigated via Metagenomic Next-Generation Sequencing. JAMA Neurol..

[23] Xing X.W., Zhang J.T., Ma Y.B., Zheng N., Yang F., Yu S.Y. (2019). Apparent performance of metagenomic next-generation sequencing in the diagnosis of cryptococcal meningitis: A descriptive study. J. Med. Microbiol..

[24] Zhang C., Wang C., Chen F., Huang Z., Fang X., Li W., Yang B., Zhang W. (2019). Metagenomic Next-Generation Sequencing Technique Helps Identify Cryptococcal Infection in the Rib: A Report of 2 Cases and Review of the Literature. JBJS Case Connect..

[25] Chen J., Li Y., Li Z., Chen G., Liu X., Ding L. (2020). Metagenomic next-generation sequencing identified Histoplasma capsulatum in the lung and epiglottis of a Chinese patient: A case report. Int. J. Infect. Dis..

[26] Dai T., Hu Q., Xie Z., Li C. (2021). Case Report: Infective Endocarditis Caused by *Aspergillus flavus* in a Hemodialysis Patient. Front. Med..

[27] He B.C., Liu L.L., Chen B.L., Zhang F., Su X. (2019). The application of next-generation sequencing in diagnosing invasive pulmonary aspergillosis: Three case reports. Am. J. Transl. Res..

[28] Alonso R., Pisa D., Aguado B., Carrasco L. (2017). Identification of Fungal Species in Brain Tissue from Alzheimer’s Disease by Next-Generation Sequencing. J. Alzheimers Dis..

[29] Alonso R., Pisa D., Carrasco L. (2019). Brain Microbiota in Huntington’s Disease Patients. Front. Microbiol..

[30] Alonso R., Pisa D., Fernandez-Fernandez A.M., Carrasco L. (2018). Infection of Fungi and Bacteria in Brain Tissue from Elderly Persons and Patients with Alzheimer’s Disease. Front. Aging Neurosci..

[31] Shivaji S., Jayasudha R., Sai Prashanthi G., Kalyana Chakravarthy S., Sharma S. (2019). The Human Ocular Surface Fungal Microbiome. Investig. Ophthalmol. Vis. Sci..

[32] Deshmukh D., Joseph J., Chakrabarti M., Sharma S., Jayasudha R., Sama K.C., Sontam B., Tyagi M., Narayanan R., Shivaji S. (2019). New insights into culture negative endophthalmitis by unbiased next generation sequencing. Sci. Rep..

[33] Imabayashi Y., Moriyama M., Takeshita T., Ieda S., Hayashida J.N., Tanaka A., Maehara T., Furukawa S., Ohta M., Kubota K. (2016). Molecular analysis of fungal populations in patients with oral candidiasis using next-generation sequencing. Sci. Rep..

[34] Mohamed N., Litlekalsoy J., Ahmed I.A., Martinsen E.M.H., Furriol J., Javier-Lopez R., Elsheikh M., Gaafar N.M., Morgado L., Mundra S. (2021). Analysis of Salivary Mycobiome in a Cohort of Oral Squamous Cell Carcinoma Patients from Sudan Identifies Higher Salivary Carriage of Malassezia as an Independent and Favorable Predictor of Overall Survival. Front. Cell Infect. Microbiol..

[35] Francoise A., Hery-Arnaud G. (2020). The Microbiome in Cystic Fibrosis Pulmonary Disease. Genes.

[36] Brunkhorst F.M., Oppert M., Marx G., Bloos F., Ludewig K., Putensen C., Nierhaus A., Jaschinski U., Meier-Hellmann A., Weyland A. (2012). Effect of empirical treatment with moxifloxacin and meropenem vs meropenem on sepsis-related organ dysfunction in patients with severe sepsis: A randomized trial. JAMA.

[37] Armstrong A.E., Rossoff J., Hollemon D., Hong D.K., Muller W.J., Chaudhury S. (2019). Cell-free DNA next-generation sequencing successfully detects infectious pathogens in pediatric oncology and hematopoietic stem cell transplant patients at risk for invasive fungal disease. Pediatr. Blood Cancer.

[38] Decker S.O., Kruger A., Wilk H., Grumaz S., Vainshtein Y., Schmitt F.C.F., Uhle F., Bruckner T., Zimmermann S., Mehrabi A. (2019). New approaches for the detection of invasive fungal diseases in patients following liver transplantation-results of an observational clinical pilot study. Langenbecks Arch. Surg..

[39] Ellis J.E., Heuser R., Missan D.S., Martinez D., Heningburg A., Shabilla M., Schwartz R., Fry S. (2017). Evidence for polymicrobial communities in explanted vascular filters and atheroma debris. Mol. Cell Probes.

[40] Mwaigwisya S., Assiri R.A., O’Grady J. (2015). Emerging commercial molecular tests for the diagnosis of bloodstream infection. Expert. Rev. Mol. Diagn..

[41] Durand C., Maubon D., Cornet M., Wang Y., Aldebert D., Garnaud C. (2021). Can We Improve Antifungal Susceptibility Testing?. Front. Cell Infect. Microbiol..

[42] Abril M.K., Barnett A.S., Wegermann K., Fountain E., Strand A., Heyman B.M., Blough B.A., Swaminathan A.C., Sharma-Kuinkel B., Ruffin F. (2016). Diagnosis of Capnocytophaga canimorsus Sepsis by Whole-Genome Next-Generation Sequencing. Open Forum Infect. Dis..

[43] De Vlaminck I., Martin L., Kertesz M., Patel K., Kowarsky M., Strehl C., Cohen G., Luikart H., Neff N.F., Okamoto J. (2015). Noninvasive monitoring of infection and rejection after lung transplantation. Proc. Natl. Acad. Sci. USA.

[44] Aubert O., Ursule-Dufait C., Brousse R., Gueguen J., Racapé M., Raynaud M., Van Loon E., Pagliazzi A., Huang E., Jordan S.C. (2024). Cell-free DNA for the detection of kidney allograft rejection. Nat. Med..

[45] Mitchell S.L., Simner P.J. (2019). Next-Generation Sequencing in Clinical Microbiology: Are We There Yet?. Clin. Lab. Med..

